# Distinct regulatory mechanisms by the nuclear Argonautes HRDE-1 and NRDE-3 in the soma of *Caenorhabditis elegans*

**DOI:** 10.1093/g3journal/jkaf057

**Published:** 2025-03-15

**Authors:** Hector Mendoza, Eshna Jash, Michael B Davis, Rebecca A Haines, Sarah VanDiepenbos, Györgyi Csankovszki

**Affiliations:** Department of Molecular, Cellular and Developmental Biology, University of Michigan, Ann Arbor, MI 48109, USA; Department of Molecular, Cellular and Developmental Biology, University of Michigan, Ann Arbor, MI 48109, USA; Department of Molecular, Cellular and Developmental Biology, University of Michigan, Ann Arbor, MI 48109, USA; Department of Molecular, Cellular and Developmental Biology, University of Michigan, Ann Arbor, MI 48109, USA; Department of Molecular, Cellular and Developmental Biology, University of Michigan, Ann Arbor, MI 48109, USA; Department of Molecular, Cellular and Developmental Biology, University of Michigan, Ann Arbor, MI 48109, USA

**Keywords:** dosage compensation, sex chromosomes, epigenetics, histone methylation, H3K9, nuclear RNAi, Argonaute, germline, soma

## Abstract

RNA interference (RNAi) is a conserved silencing mechanism that depends on the generation of small RNA molecules that leads to the degradation of the targeted messenger RNAs (mRNAs). Nuclear RNAi is a unique process that triggers regulation through epigenetic alterations to the genome. This pathway has been extensively characterized in *Caenorhabditis elegans* and involves the nuclear recruitment of H3K9 histone methyltransferases by the Argonautes HRDE-1 and NRDE-3. The coordinate regulation of genetic targets by H3K9 methylation and the nuclear Argonautes is highly complex and has been mainly described based on the small RNA populations that are involved. Recent studies have also linked the nuclear RNAi pathway to the compaction of the hermaphrodite X chromosomes during dosage compensation (DC), a mechanism that balances genetic differences between the biological sexes by repressing X chromosomes in hermaphrodites. This chromosome-wide process provides an excellent opportunity to further investigate the relationship between H3K9 methylation and the nuclear Argonautes. Our work suggests that the nuclear RNAi and the H3K9 methylation pathways each contribute to the condensation of the X chromosomes during DC but the consequences on the transcriptional output of X-linked genes are minimal. Instead, nuclear RNAi mutants exhibit global transcriptional differences, in which HRDE-1 and NRDE-3 affect expression of their mRNA targets through different relationships to H3K9 methylation.

## Introduction

RNA interference (RNAi) is a highly conserved gene silencing mechanism that primarily involves endonucleolytic degradation of RNA (Metzlaff *et al*. 1997; Maine 2000). Important discoveries within the RNAi field have been made in a variety of biological systems (Cogoni and Macino 1997; Pal-Bhadra *et al*. 1997; Fire *et al*. 1998) and more recent advances have shown the potential of RNAi technology in targeted human therapies (as reviewed in Setten *et al*. 2019). The mechanism behind RNAi-induced silencing involves the binding of small RNA molecules (sRNAs) by the RNAi-induced silencing complex, a ribonucleoprotein complex capable of targeted control of gene expression via active degradation of messenger RNA (mRNA) (Hammond *et al*. 2000; Bernstein *et al*. 2001), disruption of protein synthesis (Behm-Ansmant *et al*. 2006a, 2006b; Iwasaki *et al*. 2009), DNA elimination (Schoeberl *et al*. 2012), or epigenetic modifications (Verdel *et al*. 2004; Xie *et al*. 2004). This last type of RNAi-mediated silencing involves co-transcriptional regulation of targeted genes by the recruitment of histone methyltransferases (HMTs) and the subsequent deposition of histone 3 lysine 9 (H3K9) methylation, a highly conserved marker for heterochromatin formation (for a review, see Padeken *et al*. 2022). The overlap between RNAi and chromatin modification allows for a finer genetic control during development.

In *Caenorhabditis elegans*, RNAi-induced transcriptional silencing is referred to as nuclear RNAi. Target specificity is assured by the generation of short interfering RNAs (siRNAs) with antisense complementarity to their targets. These RNA strands are 22 nucleotides long and begin with a guanine, hence their categorization as 22G-RNAs (Gu *et al*. 2009). Worm-specific Argonaute (WAGO) proteins can then associate with and translocate 22G-RNAs to the nucleus, promote heterochromatin formation at specific genomic loci and stall transcript elongation (Guang *et al*. 2010, 2008; Buckley *et al*. 2012). This nuclear RNAi silencing pathway recruits the H3K9 HMTs MET-2 and SET-25, the H3K23 HMT SET-32, and the H3K27 HMT MES-2 (Ashe *et al*. 2012; Mao *et al*. 2015; Kalinava *et al*. 2017; Spracklin *et al*. 2017). SET-32 has also been shown to contribute to H3K9 methylation at nuclear RNAi targets (Kalinava *et al*. 2018). Recruitment of these HMTs is dependent on the WAGOs HRDE-1 and NRDE-3, which localize to the nuclei of germline (Buckley *et al*. 2012; Seroussi *et al*. 2023) and somatic cells (Guang *et al*. 2008; Seroussi *et al*. 2023), respectively.

Most of the published literature on the native targets of the nuclear WAGOs focuses on sequencing of the siRNA populations they bind. Accordingly, HRDE-1 has been associated with silencing of long terminal repeat (LTR) retrotransposons (Ni *et al*. 2014, 2018), maintenance of the germline (Buckley *et al*. 2012; Shirayama *et al*. 2012), propagation of transgenerational silencing (Buckley *et al*. 2012; Rechavi *et al*. 2014; Kim *et al*. 2021; Ding *et al*. 2023) and interplay with other RNAi silencing pathways (Bagijn *et al*. 2012; Fischer and Ruvkun 2020; Rogers and Phillips 2020). NRDE-3's targets include repetitive elements (Zhou *et al*. 2014; Padeken *et al*. 2021), ribosomal RNA (rRNA) (Zhou *et al*. 2017; Wang *et al*. 2020), intergenic regions (Zhou *et al*. 2014; Padeken *et al*. 2021), duplicated genes (Fischer *et al*. 2011), and have also been linked to the maintenance of heritable silencing (Burton *et al*. 2011) and to separate RNAi silencing mechanisms (Fischer *et al*. 2013; Zhuang *et al*. 2013). The targets of the H3K9 HMTs encompass repetitive elements (Zeller *et al*. 2016; McMurchy *et al*. 2017; Padeken *et al*. 2019) and regulators of tissue differentiation (Rechtsteiner *et al*. 2019; Methot *et al*. 2021) and developmental fate (Gonzalez-Sandoval *et al*. 2015; Bian *et al*. 2020).

The way in which the nuclear WAGOs and H3K9 methylation coordinate silencing of overlapping genetic targets is complex. For instance, H3K9 methylation is required for silencing of nuclear WAGO targets in some cases, but not others (Ashe *et al*. 2012; Kalinava *et al*. 2017; Lev *et al*. 2017; Minkina and Hunter 2017; Spracklin *et al*. 2017). Notably, in a triple *set-32; met-2  set-25* mutant background, most endogenous targets of HRDE-1 are not derepressed (Ni *et al*. 2014, 2018; Kalinava *et al*. 2017). Therefore, H3K9 methylation, specifically H3K9 trimethylation (H3K9me3), cannot be the sole mechanism of silencing directed by HRDE-1. On the other hand, cumulative derepression of LTR retrotransposons in a *hrde-1* mutant background that is also compromised for HMT activity has also been reported (Kalinava *et al*. 2018; Ni *et al*. 2018), suggesting that H3K9 methylation can contribute to silencing of nuclear WAGO targets. Previous studies on HRDE-1 focused on germline targets. However, HRDE-1 function clearly impacts somatic development, as exemplified by the derepression of transposons in intestinal cells of *met-2; hrde-1* double mutants (Ni *et al*. 2018) and the impact on X chromosome decondensation in intestinal cells in a *hrde-1* mutant background (Davis *et al*. 2022). Understanding the relationship between nuclear WAGOs and H3K9 HMTs provides a great opportunity for the exploration of how these converging silencing pathways can produce specific changes during development.

We previously showed that nuclear WAGOs and H3K9 HMTs also influence packaging of the X chromosomes during dosage compensation (DC) (Snyder *et al*. 2016; Davis *et al*. 2022), a mechanism that equalizes gene expression differences between the biological sexes. In *C. elegans*, DC targets both X chromosomes of hermaphrodites (XX), dampening their expression by ∼50% to match that of males (X0) (Meyer 2022). X-specific silencing is promoted by the enrichment of the repressive histone 4 lysine 20 (H4K20) methylation mark, mediated by the histone demethylase DPY-21 (Vielle *et al*. 2012; Wells *et al*. 2012; Brejc *et al*. 2017). Silencing of the hermaphrodite X chromosomes is reinforced by chromatin sequestration and compaction, mediated by the deposition of H3K9 methylation by MET-2, SET-25, and SET-32 (Towbin *et al*. 2012; Snyder *et al*. 2016).

DC-defective backgrounds exhibit X chromosome decondensation (Lau *et al*. 2014; Snyder *et al*. 2016; Brejc *et al*. 2017; Davis *et al*. 2022). Additionally, a wide array of phenotypes and physiological abnormalities have been previously associated with mutations affecting DC, including a shorter and stockier body (Dpy) and egg laying abnormalities (Egl) (for a comprehensive list, refer to the review by Meyer 2022) and can be accompanied by X chromosome derepression (Lau *et al*. 2014; Kramer *et al*. 2015; Snyder *et al*. 2016; Trombley *et al*. 2024). On the other hand, if ectopically activated in males, DC results in complete lethality due to the inappropriate repression of their single X chromosome (Miller *et al*. 1988). This male lethality can be rescued by mutations that affect subunits of the dosage compensation complex (DCC) (Miller *et al*. 1988; Rhind *et al*. 1995) and, to a lesser extent, by disrupting X chromosome anchoring and compaction via H3K9 methylation (Snyder *et al*. 2016) and nuclear WAGO function (Weiser *et al*. 2017; Davis *et al*. 2022). Rescue by depletion of the H3K9 HMTs or nuclear WAGOs, however, requires partial destabilization of the DC mechanism via a mutation in *sex-1*, a negative regulator of male development in the sex determination pathway of *C. elegans* (Gladden *et al*. 2007; Snyder *et al*. 2016; Davis *et al*. 2022). The potential interplay between DC, H3K9 methylation, and the nuclear WAGOs provides a unique perspective to further characterize the nuclear RNAi silencing mechanism.

In this study, we explore the impact of the loss of nuclear RNAi and/or H3K9 methylation on DC and on gene expression in the soma in general. To our knowledge, the impact of the combined loss of nuclear WAGOs and HMTs has only been investigated in the context of silencing of transposable elements by HRDE-1 (Ni *et al*. 2018). We show that H3K9 methylation and nuclear WAGOs have both overlapping and nonoverlapping functions. This finding is based on the additivity resulting from the combined action of the nuclear WAGO and the H3K9 HMTs, reflected as early developmental delays and significantly decondensed X chromosomes during DC. Analysis of transcriptomic profiles of nuclear RNAi mutants with and without additional mutations in H3K9 HMTs revealed that nuclear RNAi-mediated chromosome compaction does not result in large scale derepression of the X chromosomes. Instead, the absence of HRDE-1, NRDE-3 and the HMTs leads to gene misregulation at the global level. However, H3K9 methylation impacts HRDE-1 and NRDE-3-mediated gene regulation in strikingly different ways. While NRDE-3 and the HMTs work cooperatively, the HMTs and HRDE-1 have opposing effects on the regulation of a subset of genes, emphasizing their specific effects in somatic gene regulation.

## Materials and methods

### Nematode strains and maintenance

All nematode strains were maintained on nematode growth media (NGM) following standard protocols (Stiernagle 2006). The strains used in this study include N2 (wild-type), CSS419  *set-32 (red11)*; *met-2 (n4256) set-25 (n5021)* (Kalinava *et al*. 2017), EKM89  *hrde-1 (tm1200)*, CSS415  *set-32 (red11)*; *hrde-1 (tm1200) met-2 (n4256) set-25 (n5021)* (Kalinava *et al*. 2018), WM156  *nrde-3 (tm1116)*, and EKM205  *set-32 (red11)*; *met-2 (n4256) set-25 (n5021); nrde-3 (tm1116)*.

### Analysis of developmental rate

Synchronized L1 larval worms were obtained by bleaching gravid adults to collect embryos and then allowing them to hatch in M9 buffer (85 mM NaCl, 1 mM MgSO_4_, 22 mM KH_2_PO_4_, and 42 mM Na_2_HPO_4_) overnight. L1 animals were then plated on NGM and allowed to grow following standard protocols (Stiernagle 2006) and at 20°C. The number of L4 animals was then manually counted 42 h postfeeding. Average developmental rates were calculated from multiple biological replicates and compared using ANOVA followed by Tukey's multiple comparisons performed on GraphPad Prism v10.0 to determine statistical significance, defined as *P* < 0.05.

### RNA extraction, library preparation, and bioinformatic analysis

Total RNA was extracted from synchronized L1 worms grown on high growth media. Synchronized L1 animals were allowed a 3-h recovery period on NGM with *Escherichia coli*  OP50 as food source prior to the extraction procedure. Samples were then lysed by repeated freeze-thaw cycles and subsequent treatment with TRIzol reagent (Invitrogen). Total RNA was isolated using the RNeasy Mini Kit (QIAGEN) with on-column DNase digestion using RQ1 RNase-Free DNase (Promega). Extractions were carried out in 3 to 4 biological replicates for each strain. Poly(A)-tailed mRNA enrichment, library preparation, and next-generation sequencing were carried out in the Advanced Genomics Core at the University of Michigan. Sample concentration and quality were assessed using a Qubit fluorometer and the TapeStation (Agilent), respectively. Optimal samples (RNA Integrity Number ≥7, concentration ≥1 ng/μl) were then subjected to poly(A) enrichment using the NEBNext Poly(A) mRNA Magnetic Isolation Module (New England Biolabs). Library preparation was carried out using the NEBNext UltraExpress RNA Library Prep Kit (New England Biolabs). mRNA was fragmented and copied into first strand complementary DNA (cDNA) using reverse transcriptase and random primers. The 3′ ends of the cDNA were then adenylated and adapters were ligated. Products were purified and enriched by PCR for the generation of the final cDNA library. Quality was assessed with a Qubit fluorometer and LabChip (Perkin Elmer). Samples were then pooled and subjected to 151 bp paired-end sequencing using the NovaSeq 6000 S4 Reagent Kit and according to the manufacturer's instructions (Illumina). BCL Convert Conversion Software v4.0 (Illumina) was used to generate de-multiplexed Fastq files.

Reads were trimmed using CutAdapt (v2.3) (Martin 2011) and further evaluated with FastQC (v0.11.8) (Andrews 2010) to determine quality of the data. Reads were mapped to the reference genome Wbcel235 and read counts were generated using Bowtie 2 (v2.4.2) (Langmead and Salzberg 2012) and Htseq 2.0 (v0.13.5) (Putri *et al*. 2022). Differential gene expression analysis was performed using DESeq2 (v1.42.0) (Love *et al*. 2014). Downstream analyses were performed using R scripts and packages and only considered protein-coding transcripts. For the correlation analyses, only significantly upregulated genes (log2FC > 0 and *Padj* < 0.05) with a minimum average of the normalized count values (baseMean > 10) were considered and are included in Supplementary File 2 for reference. Gene enrichment analysis was performed using the WormBase gene set enrichment analysis tool with a *q*-value threshold of 0.1 (Angeles-Albores *et al*. 2018, 2016). Published datasets used in this study included ChIP-seq for the H3K9me3 profile in N2 (McMurchy *et al*. 2017) and smRNA-seq for validation of direct targets of both HRDE-1 and NRDE-3 (Seroussi *et al*. 2023). These data are publicly available through the NCBI Gene Expression Omnibus. Detailed protocols for the processing and downstream analyses of these datasets can be found in their original publications.

### Immunofluorescence

Immunofluorescence staining was carried out following standard protocols (Shaham 2006). Intestinal nuclei from 1-day-post L4 animals were dissected on slides in 1× sperm salts (50 mM Pipes pH 7, 25 mM KCl, 1 mM MgSO_4_, 45 mM NaCl, and 2 mM CaCl_2_), fixed in 2% paraformaldehyde, and frozen on dry ice for 10 min. Following three 10-min washes in PBS with 0.1% Triton X-100 (PBST), slides were incubated with diluted primary antibodies in a humid chamber overnight at room temperature. Slides were then washed 3 times, for 10 min each, with PBST and incubated with secondary antibodies in a humid chamber at 37°C for 1 h. Following incubation, the 3 PBST washes were repeated, with the last wash including the DAPI nuclear stain. Slides were mounted with Vectashield (Vector Labs) prior to storage at −20°C until microscopic examination.

### Fluorescence in situ hybridization combined with immunofluorescence

Slides of intestinal nuclei from 1-day-old L4 animals were prepared as for immunofluorescence up to the first set of PBST washes (see above). Slides were then dehydrated with sequential 2-minute washes in 70%, 80%, 95%, and 100% ethanol and allowed to air dry for 5 min at room temperature. Detection probes were prepared from degenerate oligonucleotide-primed PCR to amplify yeast artificial chromosomes corresponding to sections of the *C. elegans* X chromosome or chromosome I. Probes were then labeled with fluorescent dCTP-Cy3 (GE) using random priming (Prime-a-gene kit, Promega), and resuspended in Hybrisol VII (MP Biomedicals) (Csankovszki *et al*. 2004; Nabeshima *et al*. 2011; Lau *et al*. 2014; Snyder *et al*. 2016; Davis *et al*. 2022). Slides were then incubated with 10 μl of the X probe at 95°C for 5 min and then at 37°C overnight in a humid chamber. The slides were then sequentially washed at 39°C with 2× saline-sodium citrate (SSC) buffer/50% formamide for 5 min (3 washes), 2× SSC for 5 min (3 washes), and 1× SSC for 10 min (1 wash). The immunofluorescence protocol resumed after these last washes, using antibodies specific for DPY-27.

### Antibodies

Rabbit α-DPY-27 (purified antibody) (Csankovszki *et al*. 2009) was the only primary antibody used and at a 1:200 dilution. For secondary antibodies, α-rabbit Cy3 (Jackson ImmunoResearch Labs 711-165-152) and α-rabbit FITC (Jackson ImmunoResearch Labs 715-095-152) were used at 1:100 dilutions.

### Microscopy and morphometric analysis

Imaging of intestinal nuclei was achieved with a Hamamatsu ORCA-ER digital camera, mounted on an Olympus BX61 epi-fluorescence microscope with a motorized Z drive. The 60× APO oil immersion objective was used for all images, with Z stacks collected at 0.2 μm increments. All images shown correspond to projection images summed from ∼3 μm. Quantification was performed using the SlideBook 5 software (Intelligent Imaging Solutions, Denver, CO, USA). Segment masks were drawn individually for the DAPI signal and the fluorophore signals for each nucleus. These masks were established by a user-defined intensity threshold value to exclude background signal and autofluorescence. Nuclear DNA and X chromosome volumes were estimated by assigning a morphometry mask and determining their relative overlap in voxels. The chromosome territory volume was defined as the ratio of the volume of the Cy3 signal to the volume of the DAPI signal. Averages were then calculated for each mutant background analyzed and one-way ANOVA followed by Tukey's multiple comparisons was performed on GraphPad Prism v10.0 to determine statistical significance, defined as *P* < 0.05. No more than 3 nuclei were imaged from any individual animal and identical probe batches were used for each experiment with a wild-type control.

## Results

### The nuclear RNAi and H3K9 methylation machineries modulate development during the larval stages

**Fig. 1.**
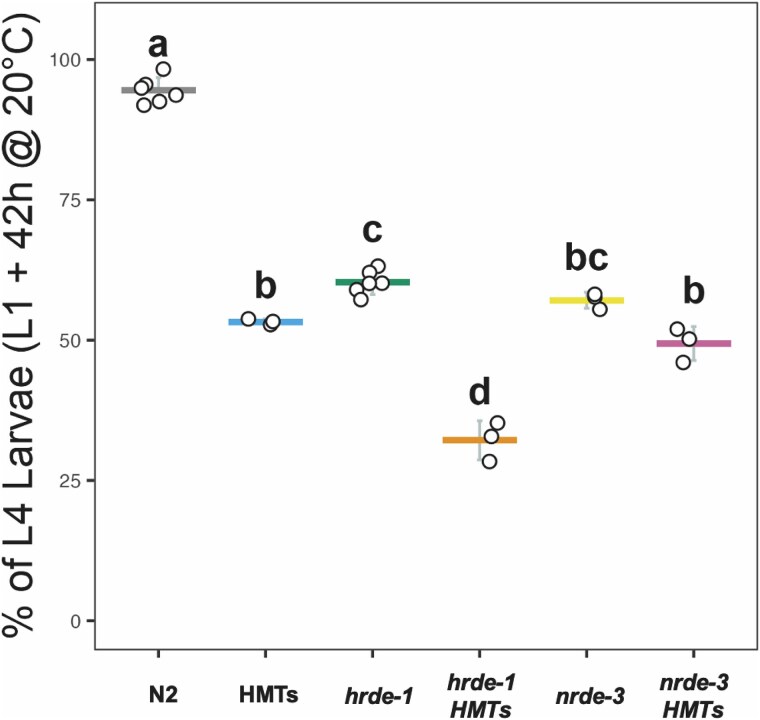
Nuclear RNAi and HMTs mutants display early developmental delay. Percentages of synchronized L1 animals that reached the L4 stage 42 h postfeeding and grown at 20°C were determined for each mutant strain. Individual points represent biological replicates, with means indicated by horizontal bars and standard deviations by vertical bars. Bars marked with the same letter show no statistically significant difference (*P* > 0.05), while those marked with different letters show statistically significant differences (*P* < 0.05). Statistical comparisons are based on one-way ANOVA followed by Tukey's multiple comparisons.

The loss of H3K9 methylation has been previously shown to delay somatic development, as *met-2  set-25* mutant animals exhibit stochastic developmental delay during the transition from the L1 stage to the L1 stage of the next generation (Zeller *et al*. 2016). We decided to investigate if the combined action of WAGO and HMTs mutations exacerbated this phenotype given that, although developmentally delayed, *met-2  set-25* mutant animals eventually reach adulthood without any noticeable aberrant morphologies (Zeller *et al*. 2016). An additional mutation was included in the *met-2  set-25* background, *set-32*, as only in its simultaneous absence can the H3K9me3 signature be fully abrogated (Kalinava *et al*. 2017, 2018). The strains studied include “HMTs” mutant (*set-32; met-2  set-25*), 2 “WAGO” mutants (*hrde-1* and *nrde-3*), and the corresponding “WAGO HMTs” combinations (*set-32; hrde-1  met-2  set-25* and *set-32; met-2  set-25; nrde-3*). We used a modified developmental assay in which synchronized L1 animals were allowed to grow under permissible conditions. The number of animals that reached the L4 stage after 42 h was then determined to examine if development was occurring at an abnormal rate. In this time frame, most of the wild-type (N2) worms reached the L4 stage (Fig. 1, gray bar). In contrast, ∼47% of HMTs mutant animals failed to reach the L4 stage at the expected time (Fig. 1, blue bar), consistent with the effect of a *met-2  set-25* mutation (Zeller *et al*. 2016). Both WAGO mutants exhibited similar developmental delays (Fig. 1, green and yellow bars). Notably, the *hrde-1* HMTs mutant background showed severe developmental delay (∼68% failed to reach the L4 stage, Fig. 1, orange bar), while the effect of the *nrde-3* HMTs combination (∼50% failed to reach the L4 stage, Fig. 1, pink bar) was comparable to that of the HMTs and *nrde-3* mutations alone. These findings corroborate the effect of H3K9 methylation in somatic development and demonstrate the additional involvement of the nuclear RNAi machinery. More importantly, the additivity exhibited in the absence of both HRDE-1 and the HMTs suggests that HRDE-1 contributes to somatic development beyond recruitment of HMTs to its targets.

### HRDE-1 contributes to X chromosome compaction during DC

Previous work suggested the involvement of the nuclear WAGOs and H3K9 HMTs in X chromosome repression during DC (Weiser *et al*. 2017; Davis *et al*. 2022), although whether they perform this function together or independently of each other is not known. To determine the impact of the combined loss of HMTs and nuclear WAGOs on X chromosome compaction, the nuclear territories occupied by both X chromosomes in hermaphrodite animals were compared between the different nuclear RNAi and HMTs mutant strains. We examined the nuclei of intestinal cells because their large size and polyploidy facilitate chromosome visualization (Hedgecock and White 1985; Fields *et al*. 2019). This methodology has been extensively used and validated in the context of *C. elegans* DC (Yonker and Meyer 2003; Csankovszki *et al*. 2004; McDonel *et al*. 2006; Lau *et al*. 2014; Snyder *et al*. 2016; Brejc *et al*. 2017). Quantification of the X chromosome territory was indirectly achieved by using antibodies against DPY-27, a protein unique to condensin I^DC^, a main component of the DCC (Chuang *et al*. 1994; Csankovszki *et al*. 2009). DPY-27 localization on the X chromosomes was confirmed via paint fluorescence in situ hybridization (FISH) combined with DPY-27 immunofluorescence, which showed that the DPY-27 signal overlaps with the X chromosome territory in all backgrounds (Supplementary Fig. 1).

**Fig. 2.**
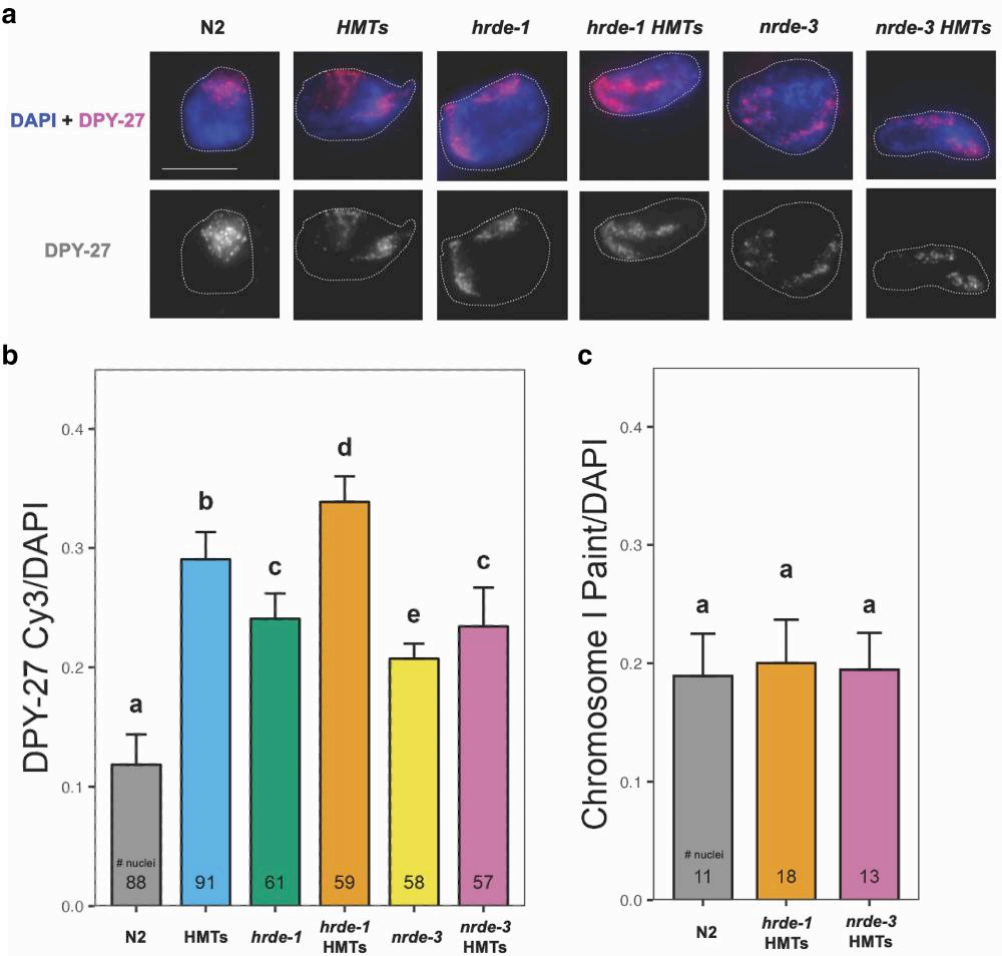
Mutations affecting the nuclear RNAi and H3K9 methylation machineries trigger X chromosome decondensation. a) Intestinal nuclei fixed from 1-day-old adult animals were subjected to DPY-27 immunofluorescent labeling using a secondary antibody labeled with Cy3 (red). No more than 3 nuclei were imaged per single animal. Labeling of total DNA was achieved using the DAPI nuclear stain (blue). The horizontal white bar represents a 10 μm scale. b) The volume of the X chromosome territory was calculated by determining the ratio of Cy3 to DAPI signal. c) For inspection of an autosome, chromosome I paint FISH (Cy3 labeled probes, red) was used. The volume of the chromosome I territory was calculated by determining the ratio of Cy3 to DAPI signal. Bars represent averages of replicates, with standard deviations and number of nuclei analyzed per strain (*n*) indicated above and within bars, respectively. Bars marked with the same letter show no statistically significant difference (*P* > 0.05), while those marked with different letters show statistically significant differences (*P* < 0.05). Statistical comparisons are based on one-way ANOVA followed by Tukey's multiple comparisons.

The X chromosome territories in the HMTs, WAGO and corresponding WAGO HMTs mutants were compared in reference to the wild-type background (Fig. 2a). X chromosome volume quantification (Fig. 2b) revealed significantly larger X chromosome territories in the HMTs mutant (blue bar) and in the nuclear WAGO mutants (green and yellow bars), consistent with previous studies (Snyder *et al*. 2016; Davis *et al*. 2022). Notably, the *hrde-1* HMTs mutant (Fig. 2b, orange bar) had a larger X chromosome territory compared to both the HMTs and the *hrde-1* mutants, suggesting an additional role for HRDE-1 in X chromosome compaction separate from the nuclear recruitment of the H3K9 HMTs. This result was not observed for the *nrde-3* HMTs mutant (Fig. 2b, pink bar), which had a reduced X chromosome territory compared to the HMTs mutant, but larger than the *nrde-3* mutant, indicating that the impacts on X chromosome packaging for these mutants are not additive. Instead, the NRDE-3 effect may be primarily attributed to H3K9 methylation and not to WAGO activity alone. A previous study reported that the nuclear WAGOs are involved in compaction of the autosomes of germ cells (Fields and Kennedy 2019). However, at least for mutations in the nuclear WAGOs or the H3K9 HMTs, this effect has not been observed in a somatic context (Snyder *et al*. 2016; Davis *et al*. 2022). We confirmed that the autosomal nuclear territory remains unchanged in the combined disruption of the nuclear WAGOs and the HMTs through chromosome I FISH (Fig. 2c). The volumes of the chromosome I territory in both WAGO HMTs mutants are comparable to the wild-type background. Thus, the compaction effect we report appears to preferentially impact the X chromosomes of somatic cells.

### Nuclear RNAi-mediated X chromosome compaction is decoupled from co-transcriptional control

**Fig. 3.**
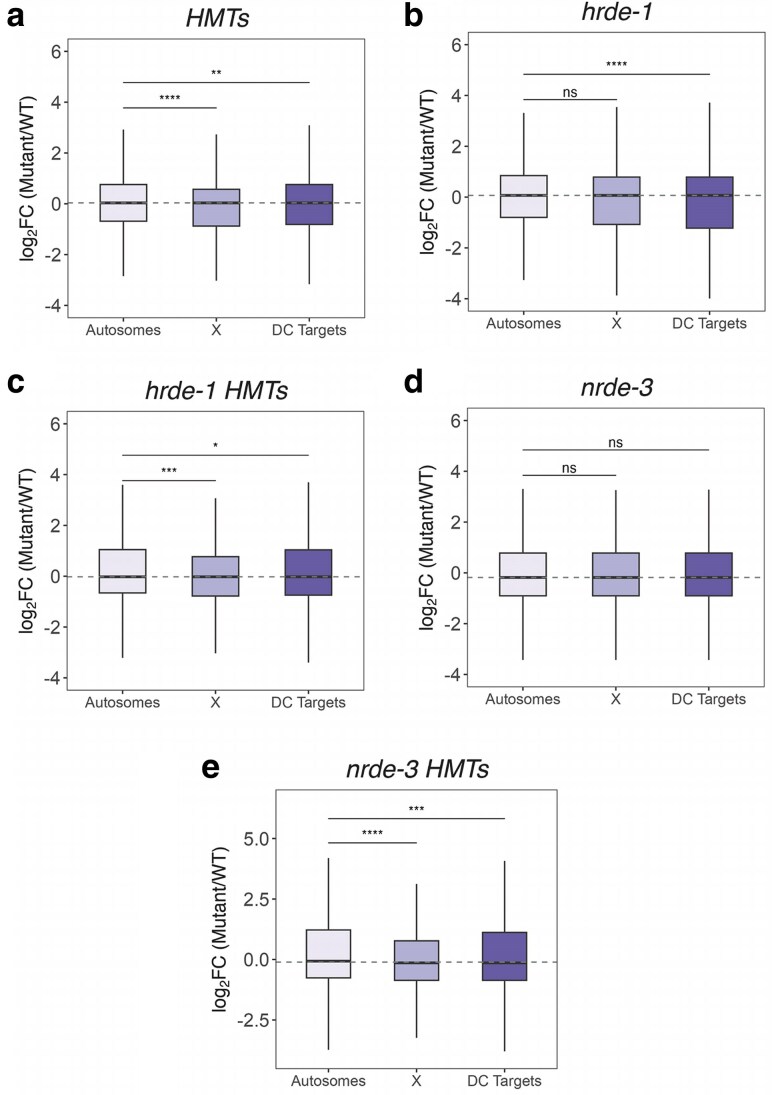
Global X chromosome expression is affected by mutations in the nuclear RNAi silencing machinery. Boxplots show the distribution of relative expression ratios (log_2_ FC) of autosomes (15653 genes), X chromosomes (2488 genes), and targets shown to be derepressed (1512 genes) in a DC-defective background based on a previous study (Snyder *et al*. 2016). Medians are indicated by solid black lines and a dashed gray line corresponding to the median of autosomes was included for better visualization of the overall expression shift. The analysis was based on differential expression of each mutant strain relative to the wild-type control: a) HMTs, b) *hrde-1*, c) *hrde-1* HMTs, d) *nrde-3*, and (e) *nrde-3* HMTs. The Wilcoxon rank-sum test was performed for statistical comparison, with asterisks indicating degree of statistical significance, defined as *P* < 0.05, shown above boxplots.

To assess the impact of nuclear RNAi and H3K9 methylation on X-linked gene expression, we used mRNA-seq to compare global chromosome expression among all autosomes and the X chromosomes in synchronized populations of L1 mutant animals in reference to the wild-type background. We used L1s because we wanted to emphasize somatic tissues, and L1 animals have only 2 germline precursor cells (for a review, see Pazdernik and Schedl 2013). Additionally, DC has been shown to begin as early as the ∼40 cell stage, (Dawes *et al*. 1999), and is well established by the L1 stage (Kramer *et al*. 2015). mRNA-seq data were first normalized based on read depth and transcript length (FPKM) and only considered protein-coding genes (18141 genes in total). We combined expression of all 5 autosomes, resulting in a total of 15,653 genes (Fig. 3, “Autosomes”). The remaining 2488 genes correspond to those on the X chromosome (Fig. 3, “X”). To more specifically look at genes that are sensitive to the disruption of DC, an additional dataset was included in the analysis. This data set was generated from synchronized populations of L1 animals treated with *dpy-27* RNAi and consisted of 1512 X-linked genes that were significantly derepressed (log2FC > 0 and *Padj* < 0.05) compared to the median autosomal gene expression change and relative to the control vector RNAi (Fig. 3, “DC Targets”) (Snyder *et al*. 2016). Relative to the combined expression of autosomal genes, X chromosome expression remained largely unaffected in all mutant backgrounds (Fig. 3a-e), indicating that X chromosome decondensation is not accompanied by significant X-linked gene derepression in these backgrounds. Although surprising, these results are consistent with the experiments assessing rescue of inappropriately dosage compensated male animals. Significant rescue upon nuclear RNAi depletion or mutation was only observed if the strain also had a sensitizing *sex-1* mutation in the background, suggesting that disruption of the nuclear RNAi machinery alone is not sufficient to significantly impact X-linked gene expression (Weiser *et al*. 2017; Davis *et al*. 2022).

### The nuclear RNAi WAGOs and H3K9 HMTs exert global gene expression control

**Fig. 4.**
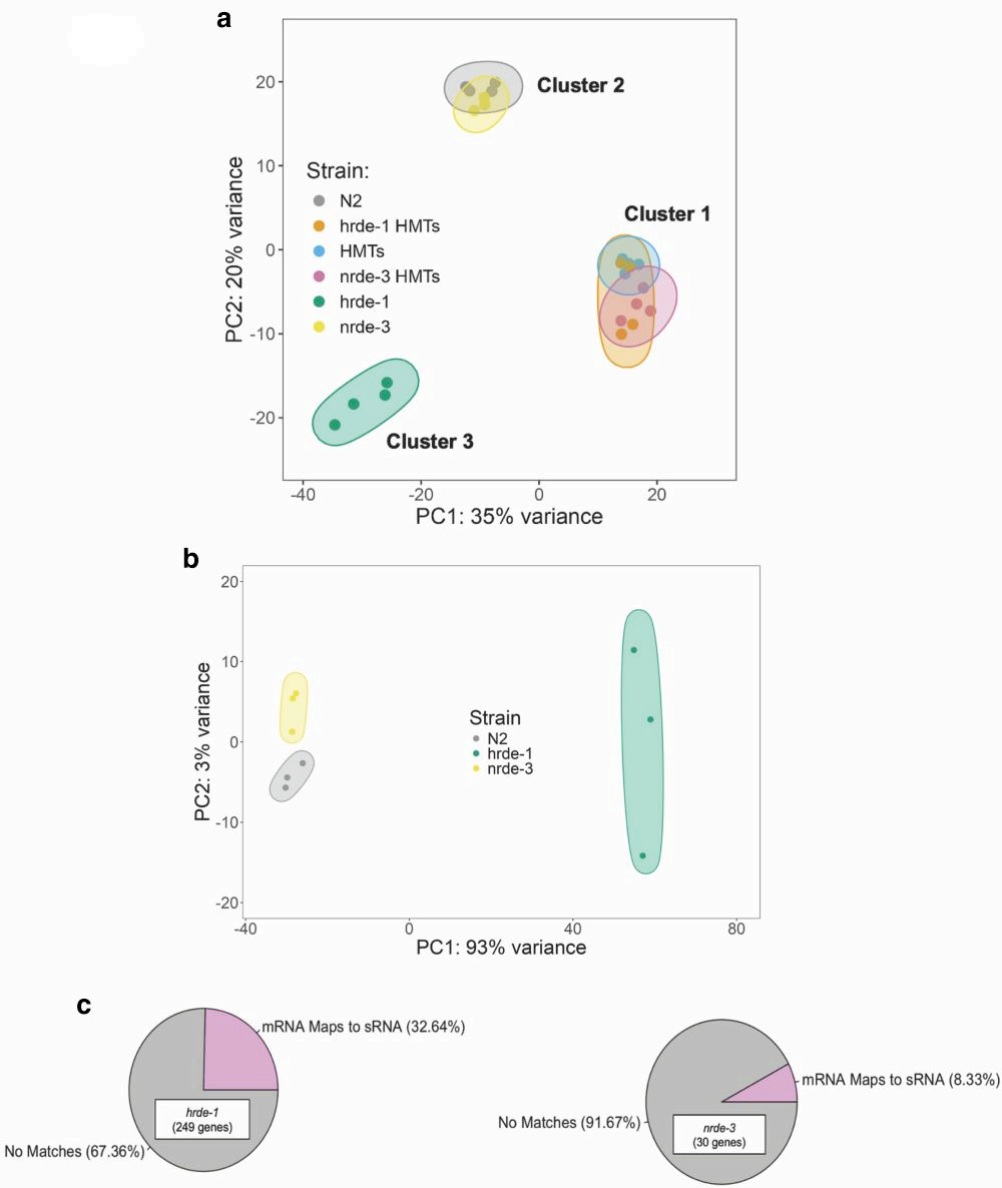
Dimensionality reduction analysis of transcriptomic data reveals distinct roles for HRDE-1 and NRDE-3. a) PCA, based on the top 500 most variable genes, of mRNA-seq data summarizing sample distribution. Individual points represent independent biological replicates for each strain analyzed. b) An additional PCA was performed on sRNA-seq data generated in a separate study (Seroussi *et al*. 2023). c) Significantly upregulated genes in our mutant/wild-type comparisons (*Padj* < 0.05, baseMean > 10) were cross-referenced with the significantly downregulated sRNA populations in *hrde-1* and *nrde-3* mutant backgrounds identified in a separate study (Seroussi *et al*. 2023).

We next assessed the effects of nuclear WAGOs and HMTs on gene expression regulation in the soma in a global context. We first used principal component analysis (PCA) to identify and rank sources of variation among all the strains analyzed. Samples with comprehensively similar transcriptomic profiles should cluster together based on the top 2 sources of variation (PC1 and PC2). Both WAGO HMTs mutants aggregated with the HMTs mutant, irrespective of the nuclear WAGO involved, indicating that gene expression changes are largely driven by the loss of H3K9 methylation (Fig. 4a, “Cluster 1”). The *nrde-3* samples grouped with the wild-type samples (Fig. 4a, “Cluster 2”), suggesting that the *nrde-3* mutation has limited impact on protein-coding gene expression and that its gene expression pattern more closely resembles the wild-type background. On the other hand, the *hrde-1* biological replicates grouped on their own, suggesting that even though HRDE-1 is a germline expressed gene, it has a significant impact on regulating gene expression in the soma (Fig. 4a, “Cluster 3”). This last trend is also in concordance with PCA performed on sRNA sequencing data (Fig. 4b) generated in a recent bioinformatic survey of *C. elegans* AGOs mutants (Seroussi *et al*. 2023), in which the *nrde-3* samples grouped near the wild-type ones, while the *hrde-1* samples clustered on their own.

We then focused on significantly upregulated genes (*Padj* < 0.05 and log2FC > 0) based on the differential expression analysis of each mutant in reference to the wild-type background. WAGO direct targets are expected to be upregulated in the mutants. We also only considered genes with a minimum mean of the normalized count values (baseMean > 10). Using these criteria, there were 249 and 30 significantly upregulated genes in the *hrde-1* and *nrde-3* mutant backgrounds, respectively. We then cross-referenced our datasets with sRNA-sequencing data. Nearly, all these genes had a corresponding sRNA based on their genomic coordinates in the wild-type background (Seroussi *et al*. 2023). We then compared these genes with genes corresponding to significantly downregulated (log2FC < 0 and *Padj* < 0.05) sRNAs in *hrde-1* and *nrde-3* mutant backgrounds (Seroussi *et al*. 2023); ∼33% of the genes upregulated in *hrde-1* mutants had corresponding HRDE-1-dependent sRNAs, and ∼8% of gene upregulated in *nrde-3* mutants had corresponding NRDE-3-dependent sRNAs (Fig. 4c). These genes represent potential direct targets of WAGOs. However, note that our samples were obtained from L1 larvae, while the sRNA data sets were generated using young adult worms, limiting this analysis.

### The nuclear WAGOs and H3K9 methylation have limited overlap on native targets

**Fig. 5.**
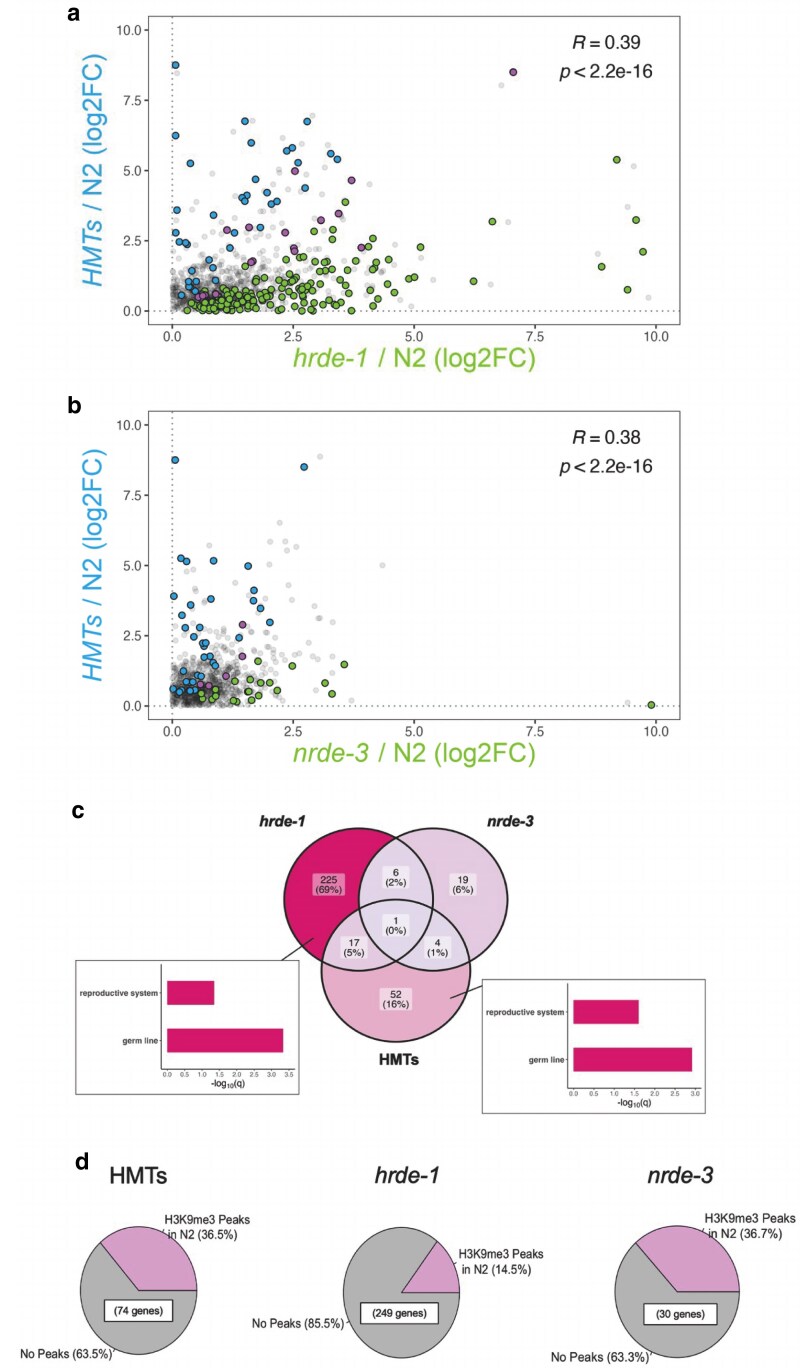
Correlation analysis between the HMTs and single WAGO mutant backgrounds. Comparisons were made between HMTs and (a) *hrde-1* or (b) *nrde-3* mutants and were based on differential expression analysis of each mutant strain relative to wild-type. Blue and green points correspond to significantly upregulated targets (log2FC > 0 and *Padj* < 0.05) with a baseMean of at least 10 in the HMTs and each WAGO mutant, respectively. Common upregulated targets are highlighted in pink, with the overall correlation computation indicated at the top-right corner. c) Downstream analysis reveals weak overlap between all 3 datasets examined, with the HMTs and HRDE-1 influencing unique gene sets significantly enriched for germline tissue (insets). d) Corroboration of our data involved cross-referencing significantly upregulated genes with those showing H3K9me3 peaks in a wild-type background, which was generated in a separate study (McMurchy *et al*. 2017).

To characterize genes regulated by HMTs, nuclear WAGOs, or both, differential expression analyses for the mutant strains against the wild-type background were filtered, defining statistical significance after multiple testing correction (*Padj* < 0.05) and a minimum mean of the normalized count values (baseMean ≥ 10) (Fig. 5a and b, colored points). We also focused on upregulated genes only to remove any indirect effects (log2FC > 0). Correlation analyses were then performed between the HMTs mutant and each WAGO mutant. For the *hrde-1*/N2 vs HMTs/N2 comparison (Fig. 5a), all genes exhibited low positive correlation (*R* ∼ 0.4). We detected 56 and 231 significantly upregulated genes in the HMTs (blue points) and the *hrde-1* (green) mutants, respectively. An additional 18 genes were significantly upregulated in both mutant backgrounds (pink points). Weak positive correlation (*R* ∼ 0.4) was also detected for the *nrde-3*/N2 vs HMTs/N2 comparison (Fig. 5b). Moreover, the HMTs and *nrde-3* mutations influenced 69 (blue points) and 25 genes (green points) on their own, respectively. Only 5 genes were affected by both mutations (pink points). Overlap between all 3 datasets was notably weak (Fig. 5c). Enrichment analysis revealed that the HMTs mutation affected a unique set of genes that were significantly enriched for germline and reproductive tissue (Fig. 5c inset and Supplementary File 1), consistent with previous findings regarding the involvement of H3K9 methylation repressing germline genes in somatic tissues (Rechtsteiner *et al*. 2019). Over half of all upregulated genes were affected by the *hrde-1* mutation alone (Fig. 5c, ∼69%), and interestingly, these genes were also significantly enriched for germline and reproductive tissue (Fig. 5c inset and Supplementary File 1). This WAGO is known to regulate genes in the germline (Buckley *et al*. 2012), but its role in regulating genes in the soma has not been characterized. The effect of NRDE-3 alone, on the other hand, is less evident, as its absence affects a much smaller gene set (Fig. 5c, ∼6%).

This first set of analyses highlight HRDE-1's influence on a gene set of considerable size (249 genes in total) that is apparently independent of H3K9 methylation. To confirm this, we cross-referenced our gene lists with H3K9me3 ChIP-sequencing data generated from wild-type young adults in a previous study (McMurchy *et al*. 2017). We used this dataset because to our knowledge, a ChIP-sequencing dataset for this heterochromatin mark that matches our larval developmental stage (L1) is not yet available. Our analysis indicates that ∼37% of the genes significantly upregulated in the HMTs mutant show H3K9me3 peaks in the wild-type background (Fig. 5d). This proportion is comparable to the *nrde-3* mutant, although the gene set in this last mutant is considerably smaller (Fig. 5d). Interestingly, only ∼15% of the genes upregulated in the *hrde-1* mutant exhibit H3K9 trimethylation, further supporting the idea that regulation of these genes by HRDE-1 may be independent of the H3K9 HMTs (Fig. 5d).

### NRDE-3 and H3K9 methylation cooperate to silence genes

**Fig. 6.**
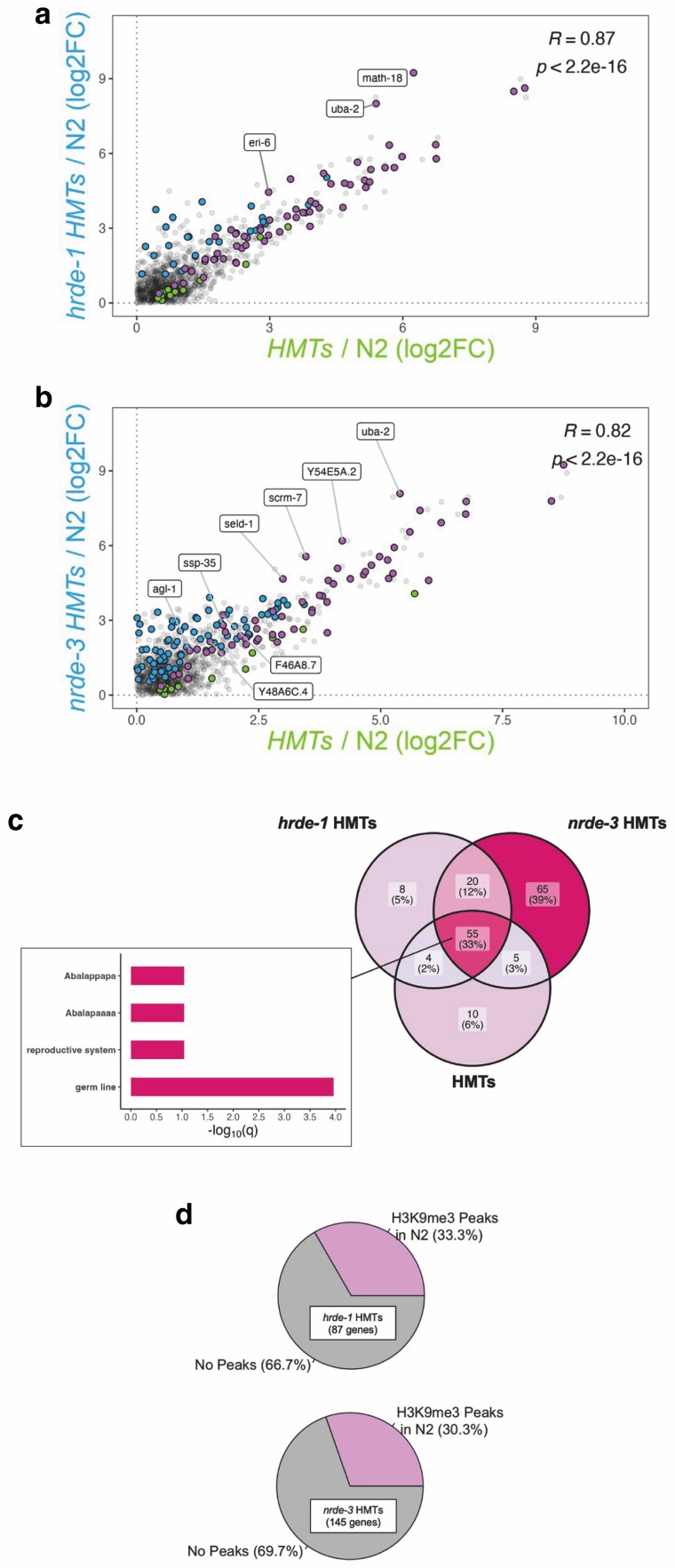
Correlation analysis between the WAGO HMTs and HMTs mutant backgrounds. Comparisons were made between HMTs and (a) *hrde-1* HMTs or (b) *nrde-3* HMTs mutants and were based on differential expression analysis of each mutant strain relative to wild-type. Blue and green points correspond to significantly upregulated targets (log2FC > 0 and *Padj* < 0.05) with a baseMean of at least 10 in the WAGO HMTs and HMTs mutants, respectively. Common upregulated targets are highlighted in pink, with the overall correlation computation indicated at the top-right corner. Labeled points correspond to genes showing true additivity (Δlog2FC > 0 and *Padj* < 0.05 in WAGO HMTs/HMTs). c) Analysis of all 3 datasets reveals significant overlap and significant germline enrichment (inset), in addition to many genes misregulated exclusively in the *nrde-3* HMTs background. d) Corroboration of our data involved cross-referencing significantly upregulated genes with those showing H3K9me3 peaks in a wild-type background, which was generated in a separate study (McMurchy *et al*. 2017).

The emergence of significantly upregulated genes in both HMTs and WAGO mutants (Fig. 5) suggests potential synergism between H3K9 methylation and nuclear WAGO function. To our knowledge, this phenomenon has only been examined in a single study and only in the context of HRDE-1 and silencing of LTR retrotransposons (Ni *et al*. 2018). Accordingly, we compared differential gene expression changes in WAGO HMTs/N2 to changes in HMTs/N2 (Fig. 6). As expected, a significant portion of genes upregulated in the HMTs background was also upregulated in the WAGO HMTs backgrounds (Fig. 6a and b, pink points). We also detected strong positive correlation (*R* ∼ 0.8–0.9), indicating that for most of these genes, the changes in expression were driven by the lack of H3K9 methylation. To determine the degree of synergism between nuclear WAGO function and H3K9 methylation, we computed the relative expression (mutant/wild-type) difference (Δlog2FC) of all common targets upregulated in both the WAGO HMTs and HMTs mutants (Fig. 6a and b, pink points). Gene with positive differences (Δlog2FC > 0, greater degree of derepression in WAGO HMT compared to HMT mutants) were then cross-referenced with differentially expressed gene sets generated by comparing the WAGO HMTs mutant against the HMTs background (WAGO HMTs/HMTs) to find statistically significant differences (*Padj* < 0.05). For HRDE-1, only 3 genes (∼5%), *eri-6*, *uba-2*, and *math-18*, exhibited true additivity based on these criteria (Fig. 6a, labeled pink points). On the other hand, and in addition to *uba-2*, 8 genes (∼12%) exhibited additivity in the context of NRDE-3 (Fig. 6b, labeled pink points). These results suggest that, unlike what was seen for HRDE-1 and LTR retrotransposons (Ni *et al*. 2018), for most genes regulated by HMTs and WAGOs, the effects of these 2 factors are not additive.

When looking at the combined gene lists, a large proportion of genes were upregulated in all 3 backgrounds (Fig. 6c, ∼33%) and this gene set was significantly enriched for germline tissue (Fig. 6c inset and Supplementary File 1). These genes are sensitive to the loss of H3K9 methylation which impacts all 3 backgrounds compared. A gene set of considerable size (Fig. 6c, ∼39%) was only upregulated in the *nrde-3* HMTs background, suggesting that NRDE-3 and the HMTs redundantly regulate these genes and that their derepression requires the loss of both H3K9 methylation and NRDE-3 function. This is in contrast with the *hrde-1* HMTs mutant background (Fig. 6c, ∼5%), suggesting that few genes are being regulated redundantly by HRDE-1 and the HMTs, accentuating different modes of regulation by the nuclear WAGOs in relation to H3K9 methylation. In these 2 last gene sets, there was no indication for germline bias after enrichment analysis, reinforcing that germline repression can be predominantly attributed to H3K9 methylation and not WAGO function alone. We confirmed this finding by cross-referencing the significantly upregulated gene sets in both WAGO HMTs mutant backgrounds with the H3K9me3 ChIP-sequencing data previously mentioned (McMurchy *et al*. 2017). Our results indicate that a significant proportion of upregulated genes (Fig. 6d, ∼33% for *hrde-1* HMTs and ∼30% for *nrde-3* HMTs) exhibit H3K9me3 in a wild-type background. These proportions are also in line with what we previously reported for the HMTs mutant (Fig. 5d).

### WAGO-mediated silencing can be enhanced by H3K9 methylation

**Fig. 7.**
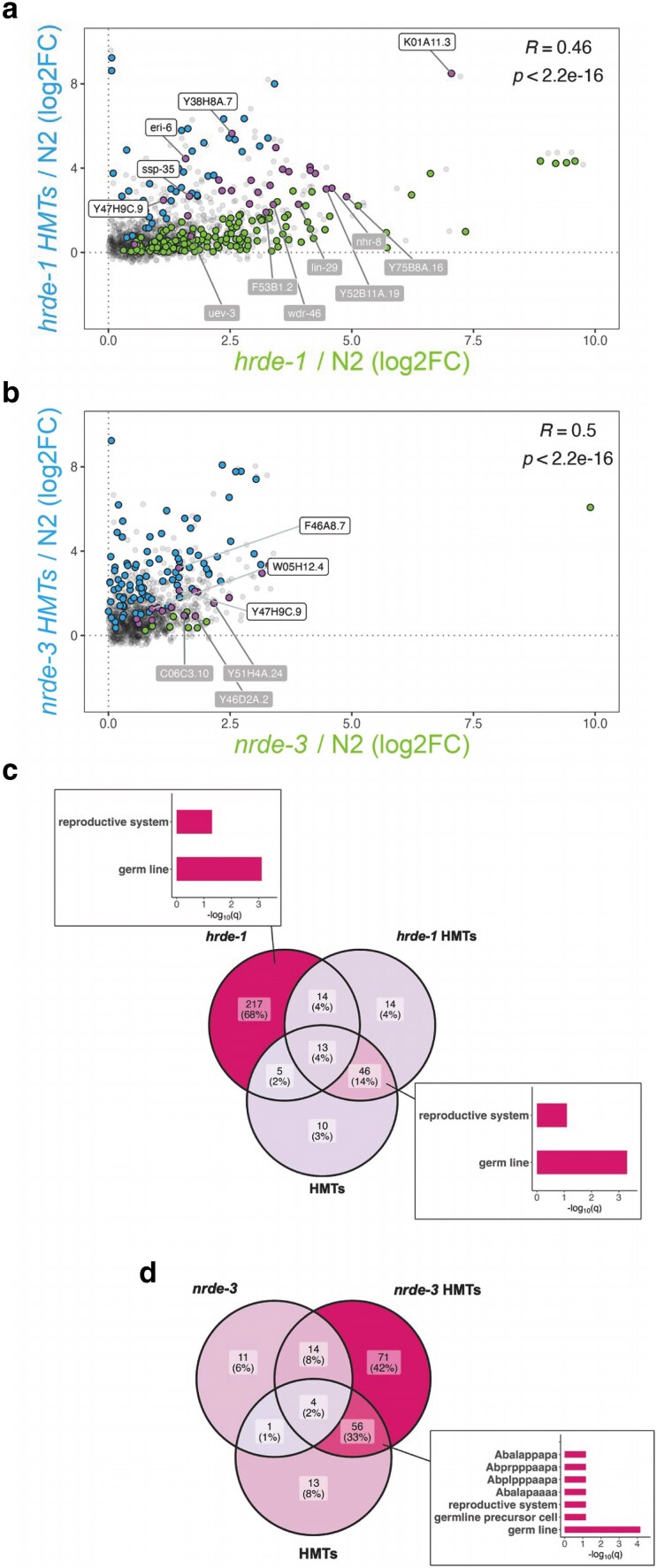
Correlation analysis between the WAGO HMTs and WAGO mutant backgrounds. Comparisons were made between the WAGO HMTs and the single (a) *hrde-1* or (b) *nrde-3* mutants and were based on differential expression analysis of each strain relative to wild-type. Blue and green points correspond to significantly upregulated targets (log2FC > 0 and *Padj* < 0.05) with a baseMean of at least 10 in the WAGO HMTs and WAGO mutants, respectively. Common upregulated targets are highlighted in pink, with the overall correlation computation indicated at the top-right corner. Points labeled in white correspond to genes showing true additivity (Δlog2FC > 0 and *Padj* < 0.05 in WAGO HMTs/HMTs), while those labeled in gray exhibit dampening (Δlog2FC < 0 and *Padj* < 0.05 in WAGO HMTs/HMTs). Overlap analysis between the WAGO, HMTs, and WAGO HMTs mutants reveals that (c) HRDE-1's predominant effect involves a mechanism separate from H3K9 methylation, while (d) NRDE-3's effect is primarily dependent on H3K9 HMT activity. Insets illustrate significant enrichment of germline-related terms.

To investigate the reverse, whether WAGO-mediated silencing can be reinforced by H3K9 methylation, we carried out correlation analyses between each WAGO HMTs mutant and the corresponding WAGO mutant. Our results indicate that many HRDE-1 targets are not upregulated in the *hrde-1* HMTs mutant (Fig. 7a, green points), suggesting that the gene expression change caused by lack of HRDE-1 was reversed by the additional loss of the HMTs. To look for potential redundancy between HRDE-1 and the HMTs, we also examined differential expression of the genes that were only upregulated in the *hrde-1* HMTs mutant alone (Fig. 7c, blue points, ∼4%). We compared gene expression levels in the *hrde-1* HMTs mutant in reference to the *hrde-1* background (*hrde-1* HMTs/*hrde-1*). Only 6 out of the 14 genes were significantly upregulated in this last comparison. These results again suggest that redundancy with the HMTs is not the main mode of regulation by HRDE-1.

Contrastingly, NRDE-3's effect seems to require H3K9 methylation, as very few targets are affected by WAGO activity alone (Fig. 7b, green points). The differences between NRDE-3 and HRDE-1 were also evident after overlap analysis, with HRDE-1 alone affecting a gene set of considerable size (∼68%) and enriched for the germline (Fig. 7c inset and Supplementary File 1). In the case of NRDE-3, more genes were affected in the *nrde-3* HMTs mutant (Fig. 7d, ∼42%). A significant proportion of these 71 genes (∼68%) were also significantly misregulated in the *nrde-3* HMTs/*nrde-3* comparison, reinforcing our previous finding that NRDE-3 and H3K9 methylation enact redundant control on their native targets. The WAGO HMTs mutants also affected genes that overlapped with the HMTs mutant (∼14% for *hrde-1* HMTs and ∼33% for *nrde-3* HMTs), which were also enriched for the germline (Fig. 7c and d insets and Supplementary File 1). This last finding suggests that the germline-specific effect in somatic tissue is primarily due to H3K9 methylation and not necessarily nuclear WAGO function.

Lastly, the additivity analyses were repeated for the common upregulated genes between the WAGO HMTs and WAGO mutants (Fig. 7a and b, pink points), this time to determine if silencing of WAGO targets is enhanced by H3K9 methylation. To determine statistical relevance, expression differences (Δlog2FC) were then cross-referenced with the WAGO HMTs/WAGO differential expression datasets. A very limited number of genes, a total of 7, exhibited significant additivity (Fig. 7a and b, pink points labeled in white). The gene *Y47H9C.9*, encoding for a protein of unknown function (Kishore *et al*. 2020), was influenced by both nuclear WAGOs. HRDE-1 alone influenced 4 genes that included *eri-6*, which re-emerged from the previous additivity analysis (Fig. 6a). In the case of NRDE-3, 1 of the 2 genes, *F46A8.7*, also appeared in the previous additivity analysis (Fig. 6b). Strikingly and unlike our previous additivity analyses, our data also indicate an antagonistic relationship (Δlog2FC < 0) between H3K9 methylation and WAGO function, (Fig. 7a and b, pink points labeled in gray), suggesting that nuclear WAGOs can target genes outside of the H3K9-mediated silencing pathway.

### HRDE-1 antagonizes H3K9 HMT-mediated repression for a subset of genes

**Fig. 8.**
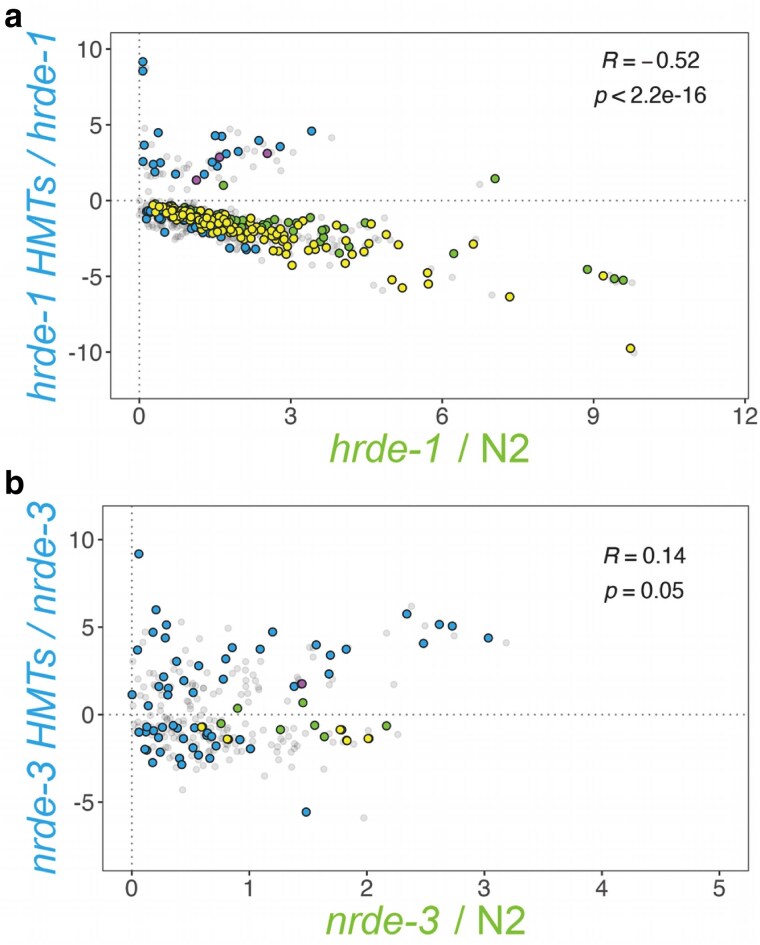
a) Correlation analysis between the *hrde-1* HMTs/HMTs and the *hrde-1*/N2 backgrounds further confirms HRDE-1's unique effect. Blue and green points correspond to significantly misregulated targets (*Padj* < 0.05) with a baseMean of at least 10 in the *hrde-1* HMTs/HMTs and *hrde-1*/N2 backgrounds, respectively. Common targets are highlighted in pink (R > 0) and yellow (R < 0), with the overall correlation computation indicated at the top-right corner. b) Correlation analysis between the *nrde-3* HMTs/*nrde-3* and *nrde-3*/N2 backgrounds. Blue and green points correspond to significantly misregulated targets (*Padj* < 0.05) with a baseMean of at least 10 in the *nrde-3* HMTs/nrde-3 and *nrde-*3/N2 backgrounds, respectively. Common targets are highlighted in pink (R > 0) and yellow (R < 0), with the overall correlation computation indicated at the top-right corner.

According to the proposed model based on the existing literature, the nuclear WAGOs recruit the H3K9 HMTs to specific gene loci to deposit the respective heterochromatin marks (Guang *et al*. 2010; Ashe *et al*. 2012; Ni *et al*. 2014; Kalinava *et al*. 2018, 2017; Kim *et al*. 2021). In our analysis, over 200 genes exhibit significant upregulation in the *hrde-1* mutant, but not in the HMTs mutants (Fig. 5a and c). Many of these genes remain unaffected in the *hrde-1* HMTs mutant background (Fig. 7a and c), leading us to question the relationship between HRDE-1 and H3K9 methylation for these genes. Additionally, for a limited set of genes, there seems to be an antagonistic relationship between the HMTs and HRDE-1 (Fig. 7a, pink points labeled in gray). This finding has been previously reported but only in the context of *hrde-1* and a *met-2  set-25* double mutant and their impact on heritable RNAi (Lev *et al*. 2017; Zhebrun *et al*. 2025). To examine this in more detail, we performed correlation analyses between *hrde-1*/N2 and *hrde-1* HMTs/*hrde-1*, with the latter defining the HRDE-1-independent HMTs effect (Fig. 8a). This analysis revealed that the HRDE-1-independent HMTs effect does not produce significant changes in gene expression on its own (Fig. 8a, blue points). Instead, most of the genes are significantly misregulated in both comparisons (Fig. 8a, yellow points) and are strongly anticorrelated (*R* ∼ −0.5). Our data suggests that genes regulated by HRDE-1 in the wild type background are also regulated by the HMTs in the absence of HRDE-1, but in the opposite direction. This trend was not evident in the context of NRDE-3 (Fig. 8b).

## Discussion

### Nuclear RNAi as a supporting mechanism for DC

We previously showed that H3K9 methylation and nuclear WAGOs have an impact on X chromosome compaction and DC (Snyder *et al*. 2016; Davis *et al*. 2022). In this study, we show that nuclear RNAi and H3K9 methylation mainly impact DC at the level of X chromatin remodeling (Fig. 2), with only minimal changes in overall X-linked gene expression in an otherwise wild-type background (Fig. 3). H3K9 methylation is a conserved epigenetic modification found predominantly on silent chromatin (Zhou *et al*. 2011), although active transcription of genomic regions enriched for H3K9 methylation has been previously reported in *C. elegans* (Kalinava *et al*. 2017; Methot *et al*. 2021) and in other model systems (Vakoc *et al*. 2005; Kim *et al*. 2007). It is also important to note that our HMTs mutant included disruption of *set-32*. Previous studies indicated that SET-32 promotes H3K9me3, and complete loss of H3K9me3 requires the *set-32* mutation, in addition to mutations in *met-2* and *set-25* (Kalinava *et al*. 2018). More recently, SET-32 was shown to be responsible for the deposition of H3K23me3, another heterochromatin mark (Schwartz-Orbach *et al*. 2020). A more recent study identified a second H3K23 HMT, SET-21, which is thought to cooperate with SET-32 for the establishment of heritable germline silencing (Zhebrun *et al*. 2025). We do not know to what extent H3K23me3 is impacted in our HMTs strain, and we also do not know how much changes in H3K23 methylation impacted gene regulation and DC, an important future line of investigation.

The limited impact on the transcriptional output of the X chromosomes is consistent with the finding that mutating or depleting the nuclear WAGOs or the H3K9 HMTs in males in which DC is inappropriately turned on results in rescue, but only in a sensitized background that partially disrupts DC (Snyder *et al*. 2016; Weiser *et al*. 2017; Davis *et al*. 2022). Specifically, this rescue requires a mutation in *sex-1*, a negative regulator of male development (Davis *et al*. 2022). Our results indicate that without this sensitizer mutation in the background, X-linked genes are not derepressed when WAGOs and/or the H3K9 HMTs are disrupted. Accordingly, X-linked gene derepression may be detected in a mutant background in which disruption of DC is achieved by targeting both *sex-1* and HMTs or nuclear WAGOs.

H3K9 methylation mediates the packaging of the genome within the nucleus and it works with CEC-4, a nuclear envelope protein that binds to H3K9 methylation marks to anchor the chromosome arms to the nuclear periphery and form active and inactive genome compartments (Gonzalez-Sandoval *et al*. 2015; Bian *et al*. 2020). The lack of CEC-4 function alone does not have major impacts on X-linked gene expression, although hermaphrodites do become more sensitive to either the depletion of DPY-27 or a *dpy-21* mutation (Trombley *et al*. 2024). These observations reveal the involvement of different supplemental mechanisms that support DC, and that loss of any single mechanism may not be enough to significantly disrupt DC and lead to measurable loss of X-linked repression.

### The interplay between H3K9 methylation and nuclear WAGO activity

We looked for evidence of synergism between H3K9 methylation and nuclear WAGO function. This analysis allowed us to determine that silencing of H3K9 methylation targets exhibit limited amplification by both nuclear WAGOs (Fig. 6). Specifically, we identified 10 truly additive targets, of which one, *uba-2*, was affected by both nuclear WAGOs. This gene encodes for one of the heterodimeric subunits of a ubiquitin-activating enzyme (E1) involved in the charging of SMO-1, the only small ubiquitin like modifier (SUMO) found in *C. elegans* (Choudhury and Li 1997; Surana *et al*. 2017). Previous RNAi experiments showed that depletion of SMO-1 leads to embryonic arrest (Fraser *et al*. 2000). Subsequent studies demonstrated that the process of SUMOylation itself is an essential post-translational modification during cellular division and cell cycle progression (Pelisch *et al*. 2014; Tsur *et al*. 2015) in a variety of tissues that include the germline (Kaminsky *et al*. 2009). Defects in this process could contribute to the early developmental delay we detected in the nuclear RNAi mutants (Fig. 1).

The *eri-6* and *math-18* exhibited augmented silencing in the context of HRDE-1. Notably, *eri-6* encodes for a component of the enhanced (Eri) RNAi pathway (Fischer *et al*. 2008), suggesting biological crosstalk between 2 separate RNAi silencing mechanisms. This observation is also consistent with other findings, in which disruption of HRDE-1 was associated with abnormal mRNA levels of *eri-6* (Ni *et al*. 2014; Rogers and Phillips 2020). In contrast, NRDE-3 enhanced silencing of 7 genes, of which *F46A8.7*, enriched in neural tissue and involved in *daf-2*-dependent longevity (Ruzanov *et al*. 2007; Kishore *et al*. 2020), *Y48A6C.4*, encoding a putative component of the MML1 complex, which is crucial during larval development (Kishore *et al*. 2020), and *ssp-35*, a spermatogenic gene (Kishore *et al*. 2020) are worth paying attention to due to the developmental delay and the overall impact on germline genes detected in this study.

We then wondered if nuclear WAGO targets also experience additive silencing by H3K9 methylation (Fig. 7). A total of 8 genes exhibited amplified silencing, with *Y47H9C.9* influenced by either nuclear WAGO. The *eri*-6 and *ssp-35* reappeared in the context of HRDE-1 only. Interestingly, these correlations also revealed an antagonistic relationship between the nuclear WAGOs and H3K9 methylation (Fig. 7a and b, pink points labeled in gray).

### Distinct regulatory mechanisms by the nuclear Argonautes and H3K9 methylation

While synergism between nuclear WAGO function and HMTs was only seen for a limited number of genes, our global transcriptomic analyses revealed fundamentally different mechanisms of regulation for a larger set of genes. Most of the genes regulated by NRDE-3 are redundantly regulated by NRDE-3 and H3K9 methylation. These genes are only misregulated in worms lacking both the HMTs and NRDE-3 (Figs. 6c and 7d). This was also corroborated by examining the H3K9me3 profiles in a wild-type background (Figs. 5d and 6d). By contrast, very few genes are regulated redundantly by the HMTs and HRDE-1 (Figs. 5c and 7c). Instead, the majority of HRDE-1 regulated genes are only misregulated in the *hrde-1* mutant background, and for many their expression does not change in the HTMs background. This last finding was also reflected in a lower proportion of misregulated genes exhibiting H3K9me3 peaks in a wild-type background (Fig. 5d). Furthermore, many of these genes are no longer misregulated in the *hrde-1* HMTs background (Fig. 7a and c), indicating an antagonistic effect between HRDE-1 and H3K9 methylation. These results are different from the mechanism behind nuclear HRDE-1 regulation of noncoding targets like the Cer3 and Cer8 retrotransposons in the germline. For repression of these targets, HRDE-1 function is essential, while H3K9 methylation is dispensable but can synergistically function with HRDE-1 to significantly increase the level of misregulation (Ni *et al*. 2018). The differences imply either a fundamental difference between the regulation of protein-coding genes and regulation of transposons, or a difference between the impact of HRDE-1's function in the soma vs the germline.

## Supplementary Material

jkaf057_Supplementary_Data

## Data Availability

RNA-seq datasets generated in this study are available at the NCBI Gene Expression Omnibus database, accession GSE277005. Datasets previously published and used in this study are also available at the NCBI GEO database, accessions GSE208702 (sRNA-seq, see Seroussi *et al*. 2023) and GSE87524 (ChIP-seq, see McMurchy *et al*. 2017). Supplemental material available at G3 online.
