# Supplementary material for: Distinct regulatory mechanisms by the nuclear Argonautes HRDE-1 and NRDE-3 in the soma of Caenorhabditis elegans
Source: G3 (Bethesda). 2025 Mar 15;15(5):jkaf057. doi: 10.1093/g3journal/jkaf057 (PMC12060244; doi:10.1093/g3journal/jkaf057)
Supplement: jkaf057_Supplementary_Data [file jkaf057_supplementary_data.zip › Supplemental_Material_Legends_G3-2025-405753.docx]

**Figure S1.** Maximum intensity projections of three-dimensional image stacks of X chromosome territories. **(A)** Intestinal nuclei fixed from one-day-old adult animals were subjected to X chromosome paint fluorescence *in situ* hybridization (Cy3 labelled probes, green). Subsequent immunofluorescent labelling was carried out using α-DPY-27 antibodies and FITC-conjugated secondary antibodies (red). Labelling of total DNA was achieved using the DAPI nuclear stain (blue). The horizontal white bar represents a 10 μm scale. **(B)** Nuclear DNA, DPY-27 and X chromosome territories were identified by assigning a corresponding morphometry mask (voxels) and determining relative overlap.

**File S1.** Raw data for enrichment analyses related to Figures 5 – 7. Analyses were performed using the WormBase gene set enrichment analysis tool with a *q*-value threshold of 0.1 (<https://wormbase.org/tools/enrichment/tea/tea.cgi>).

**File S2.** Differential expression (mutant/N2) datasets filtered according to statistical significance (*padj* < 0.05) and the average of the normalized count values (baseMean > 10).
