# Supplementary figures and images for: Distinct regulatory mechanisms by the nuclear Argonautes HRDE-1 and NRDE-3 in the soma of Caenorhabditis elegans
Source: G3 (Bethesda). 2025 Mar 15;15(5):jkaf057. doi: 10.1093/g3journal/jkaf057 (PMC12060244; doi:10.1093/g3journal/jkaf057)

FIGURE S1

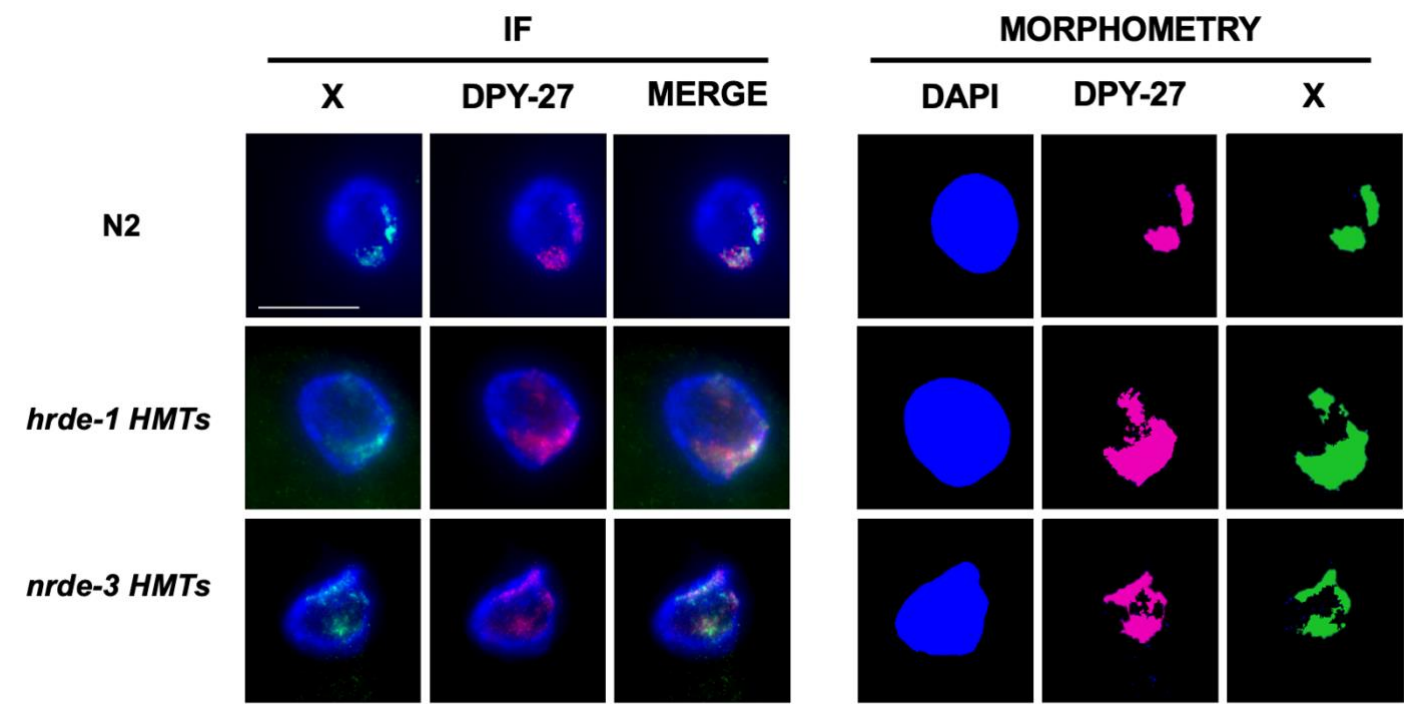

Supplement: jkaf057_Supplementary_Data [file jkaf057_supplementary_data.zip › Figure_S1_G3-2025-405753.pdf]
